# Supplementary figures and images for: Breaking the reproducibility barrier with standardized protocols for plant–microbiome research
Source: PLoS Biol. 2025 Sep 8;23(9):e3003358. doi: 10.1371/journal.pbio.3003358 (PMC12416739; doi:10.1371/journal.pbio.3003358)

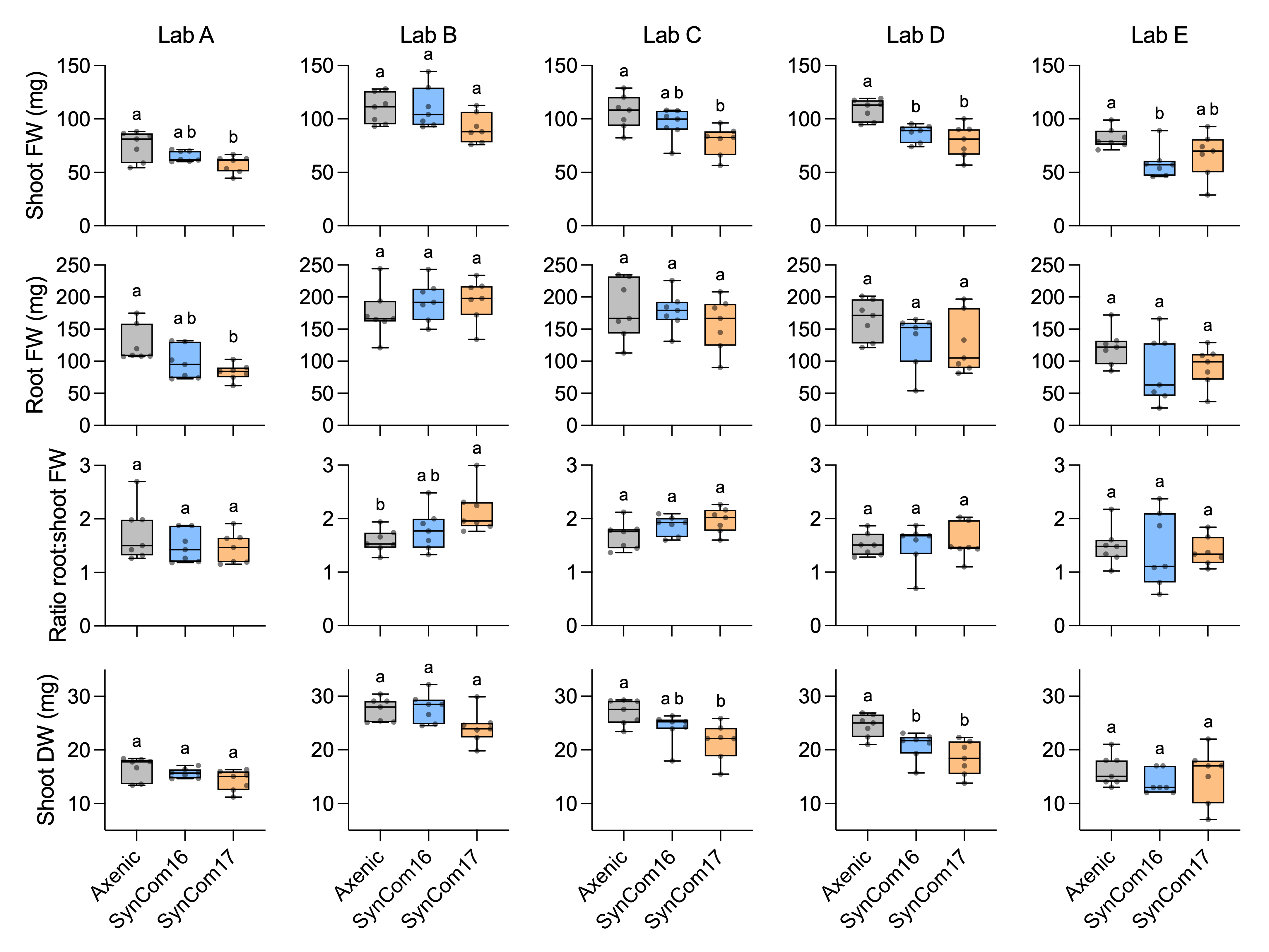

Supplement: S1 Fig — Box plots display all data points, with hinges spanning the 25th to 75th percentiles, a central line denoting the median, and whiskers reaching the minimum and maximum values. Different lowercase letters indicate statistically significant differences at p < 0.05. One-way ANOVA with Tukey test (n = 7). The shoot photos can be found at https://doi.org/10.6084/m9.figshare.26409310. The data underlying this figure can be found at https://doi.org/10.6084/m9.figshare.26401315. (TIFF) [file pbio.3003358.s001.tiff]

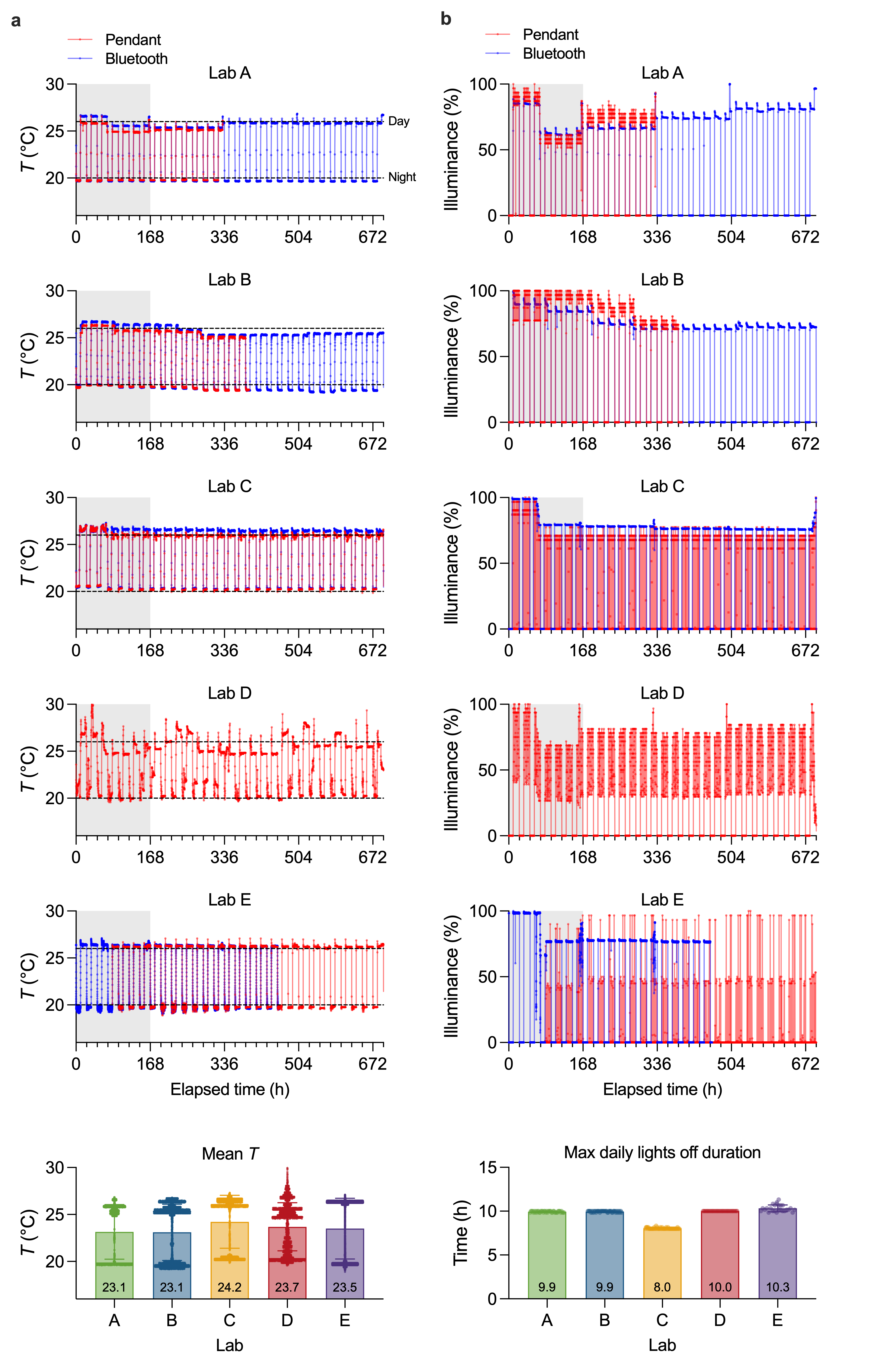

Supplement: S2 Fig — (a) Temperature (T) and (b) Normalized illuminance (% of lab maximum value) to assess lights-off duration, measured by HOBO loggers (Pendant model #UA-00264 in red, Bluetooth model #MX2202 in blue). Dashed lines show the set day/night T (26/20 °C); gray areas mark the 7-day pre-inoculation period. Labs A, B, and E experienced logging interruptions due to battery drainage; Lab D’s Bluetooth logger did not cover the experimental period. The data underlying this figure can be found at https://doi.org/10.6084/m9.figshare.26401315. (TIFF) [file pbio.3003358.s002.tiff]

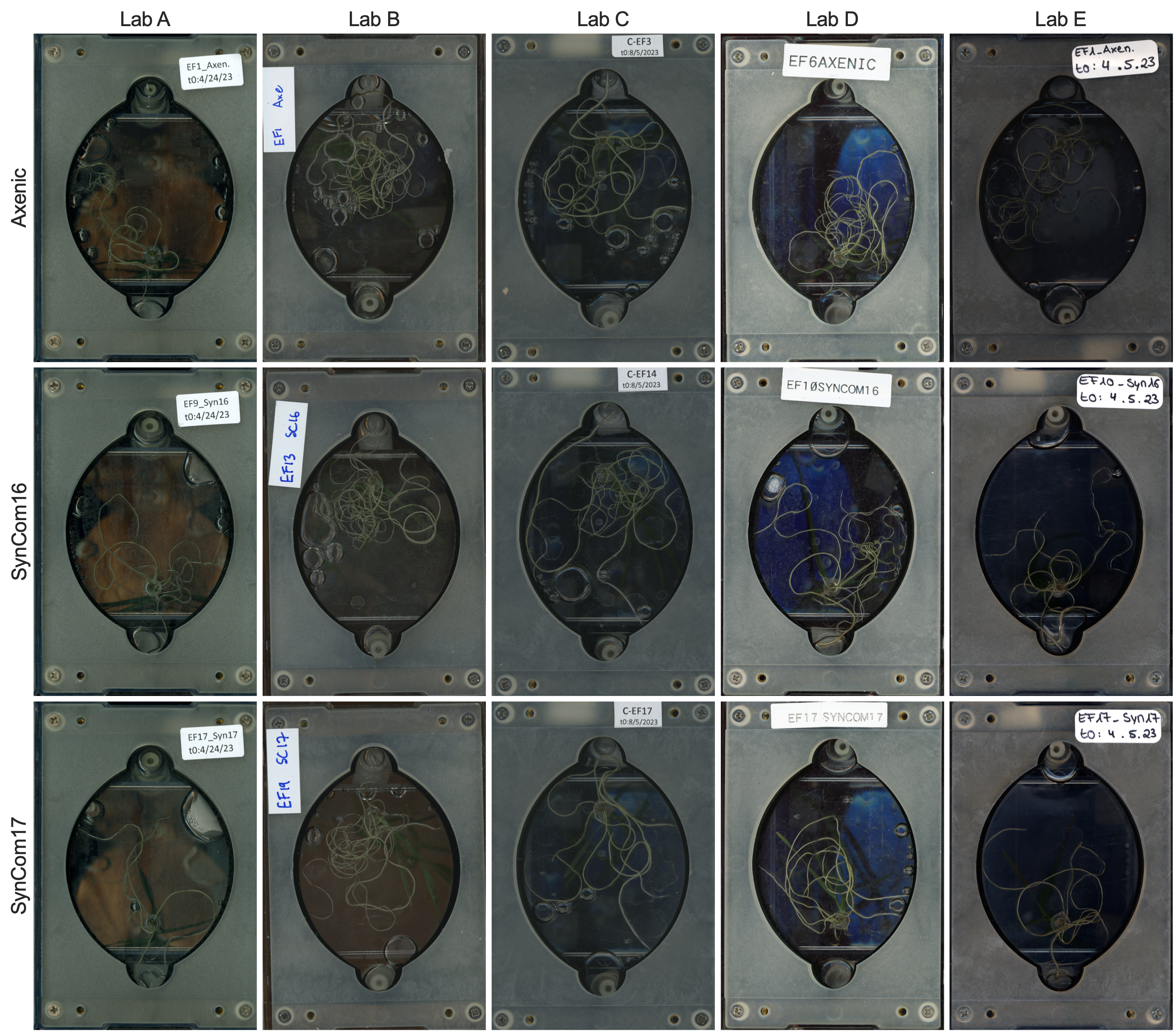

Supplement: S3 Fig — Labs A–E imaged roots in EcoFAB 2.0 devices with flatbed scanners. The figure shows plants at harvest (22 DAI). The root scans and Rhizonet reports can be found at https://doi.org/10.6084/m9.figshare.26131291. The data underlying this figure can be found at https://doi.org/10.6084/m9.figshare.26401315. (TIFF) [file pbio.3003358.s003.tiff]

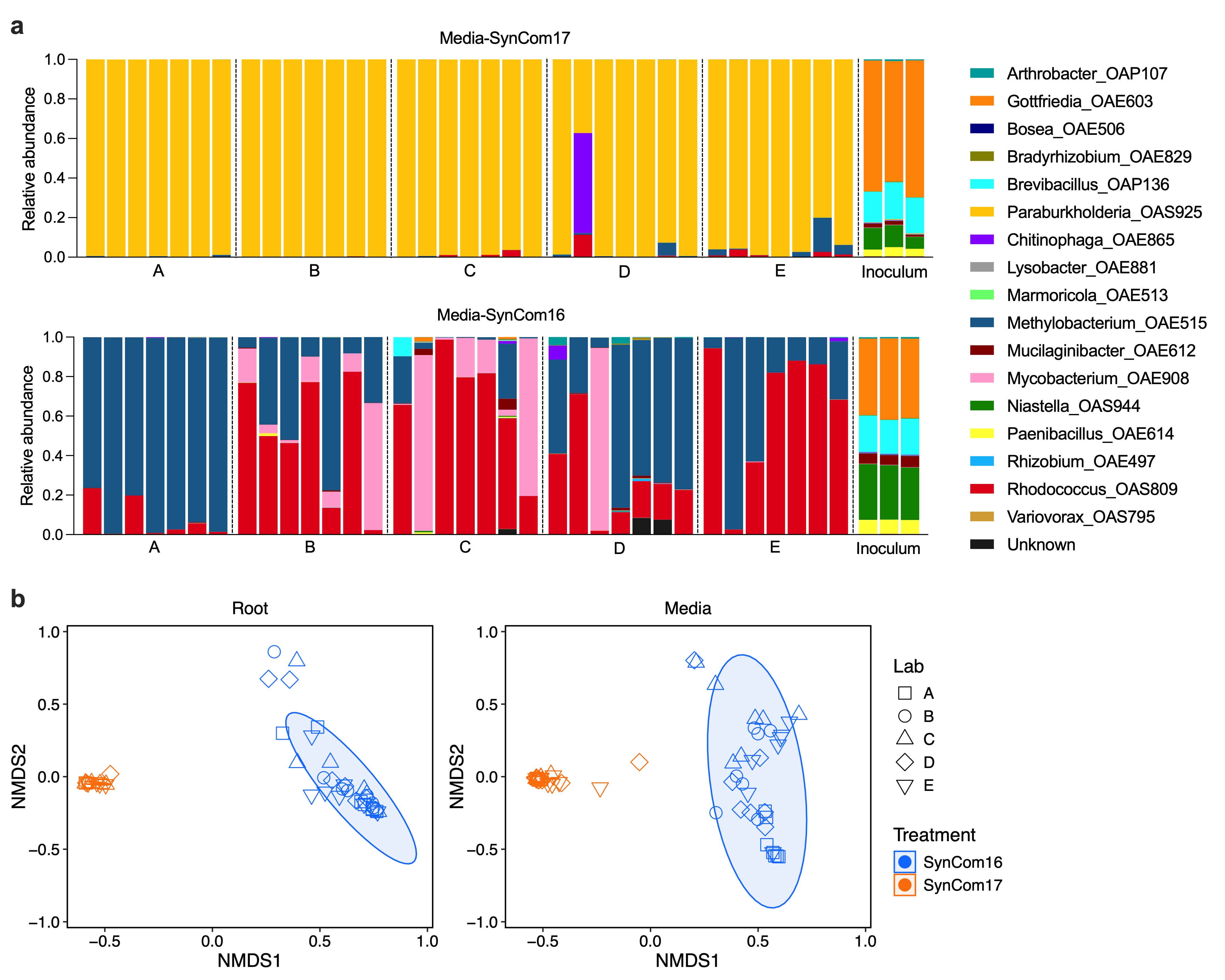

Supplement: S4 Fig — (a) Microbiome in plant growth media and starting inoculum. Letters indicate different laboratories, with each biological replicate shown (n = 7). The inoculum shows technical replicates (n = 3). (b) NMDS plot of root and media microbiomes with 95% confidence ellipse. Different laboratories are shown with various symbols, while colors represent SynCom16 (blue) versus SynCom17 (orange) inoculated plants. The data underlying this figure can be found at https://doi.org/10.6084/m9.figshare.26401315 or in S3 Table. (TIFF) [file pbio.3003358.s004.tiff]

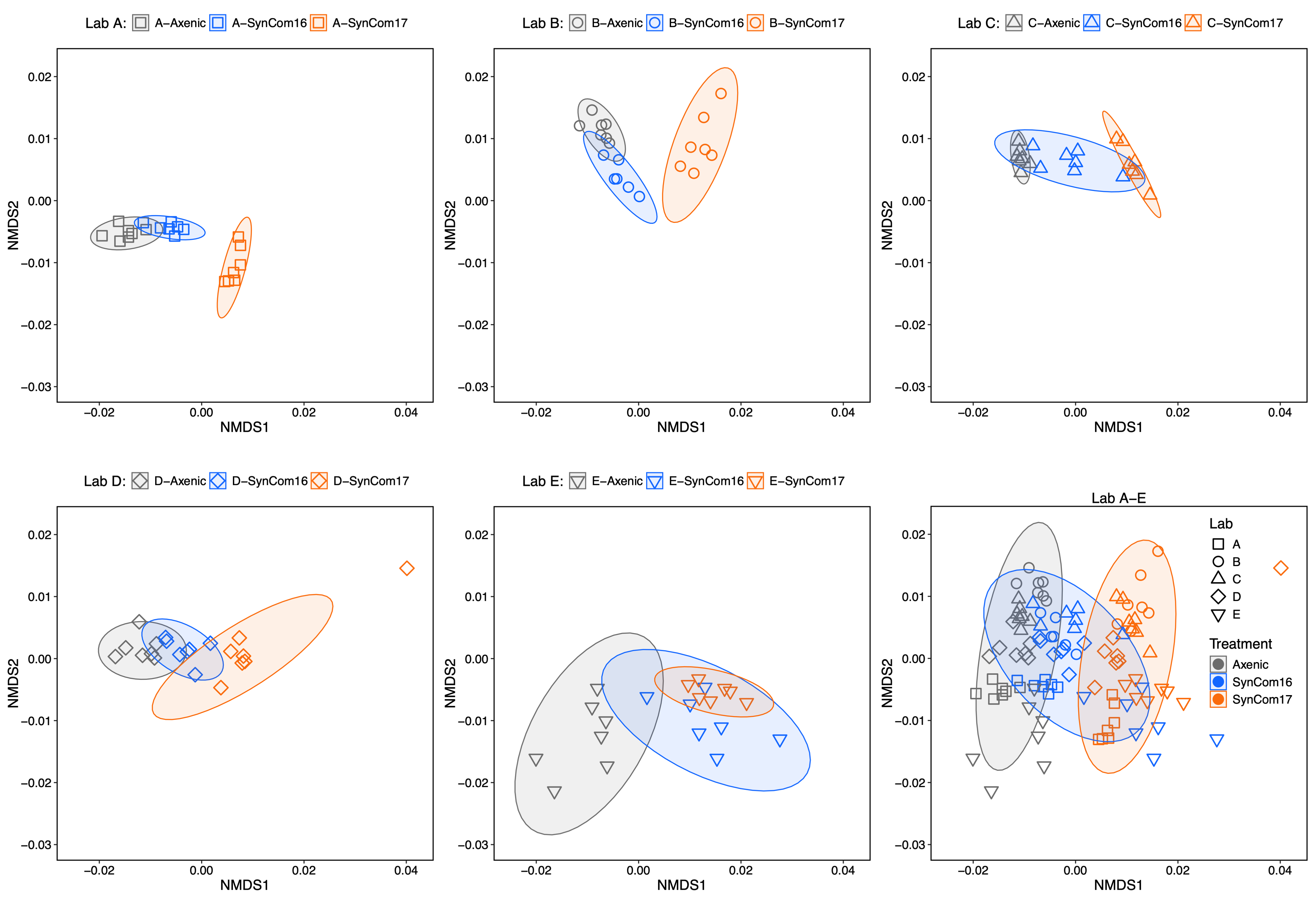

Supplement: S5 Fig — NMDS plots with a 95% confidence ellipse for 833 filter features for individual laboratories A–E and all combined. Different colors show treatments: Axenic (gray), SynCom16 (blue), and SynCom17 (orange), while shapes indicate laboratories in the combined plot. The data underlying this figure can be found at https://doi.org/10.6084/m9.figshare.26401315. (TIFF) [file pbio.3003358.s005.tiff]

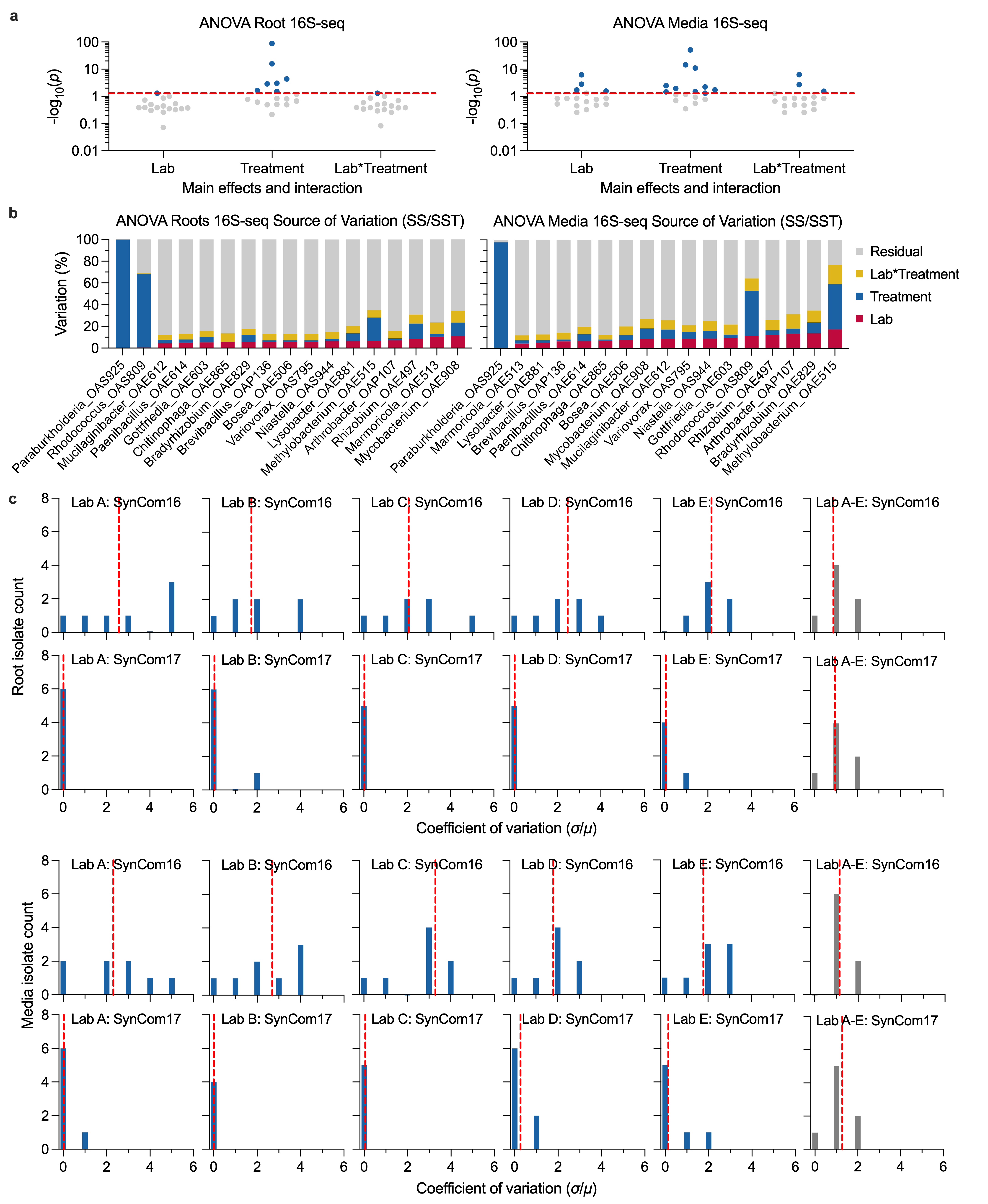

Supplement: S6 Fig — (a) ANOVA p-value of effects for root and media. Each point is a SynCom member with blue indicating significant values (red line p = 0.05). (b) ANOVA source of variation for root and media calculated from ratio of squares (SS) for each effect and total sum of squares (TSS). (c) Coefficient of variation (CV = σ/μ) distribution (median in red) for treatments within (blue) and across (gray) labs for root and media. The data underlying this figure can be found at https://doi.org/10.6084/m9.figshare.26401315. (TIFF) [file pbio.3003358.s006.tiff]

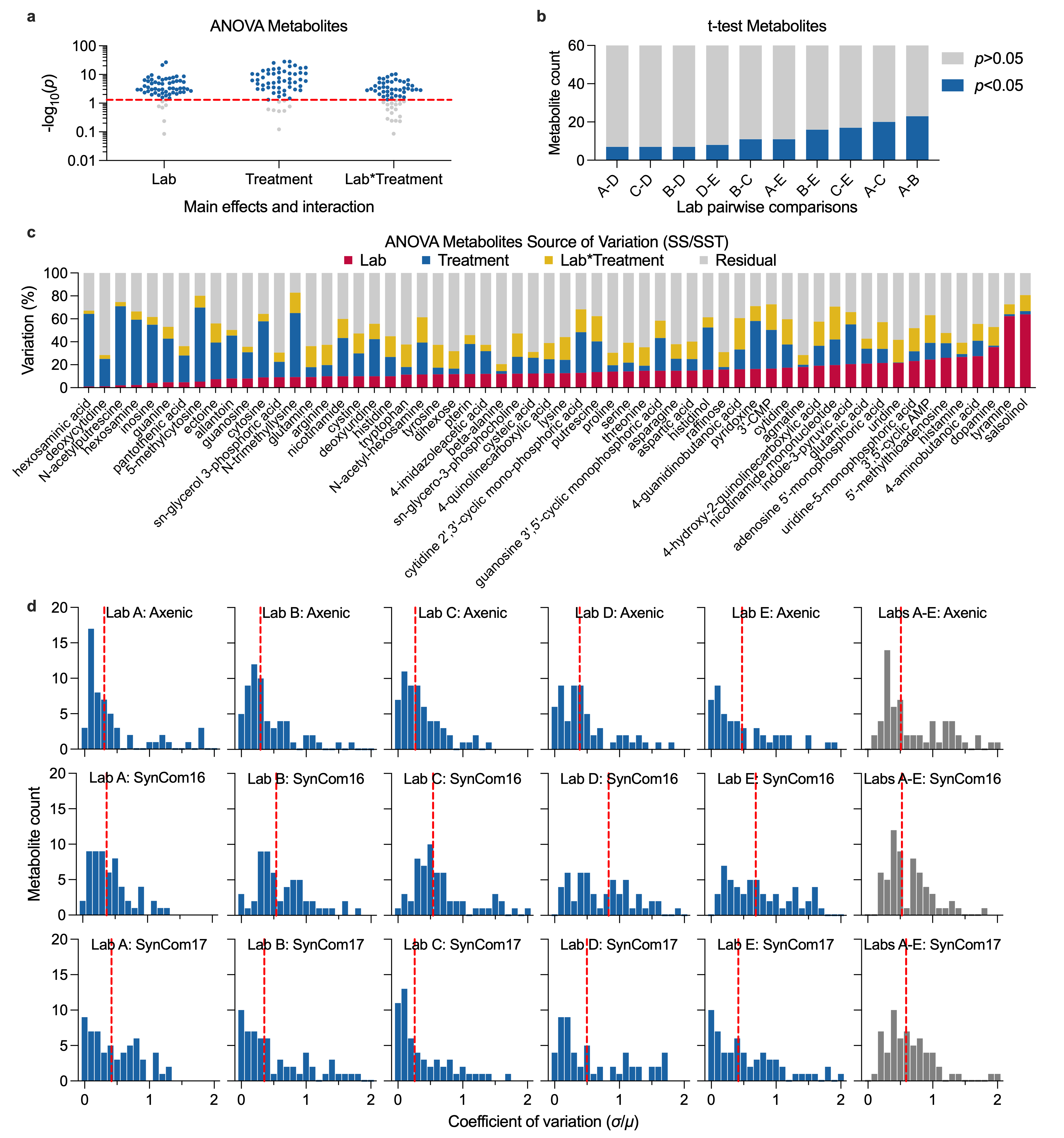

Supplement: S7 Fig — (a) ANOVA p-value of effects for metabolite peak heights. Each point is a metabolite with blue indicating significant values (red line p = 0.05). (b) Number of statistically different metabolites (p < 0.05) in lab pairwise comparisons. (c) ANOVA source of variation for root and media calculated from ratio of squares (SS) for each effect and total sum of squares (TSS). (d) Coefficient of variation (CV = σ/μ) distribution (median in red) for treatments within (blue) and across (gray) labs. The data underlying this figure can be found at https://doi.org/10.6084/m9.figshare.26401315. (TIFF) [file pbio.3003358.s007.tiff]

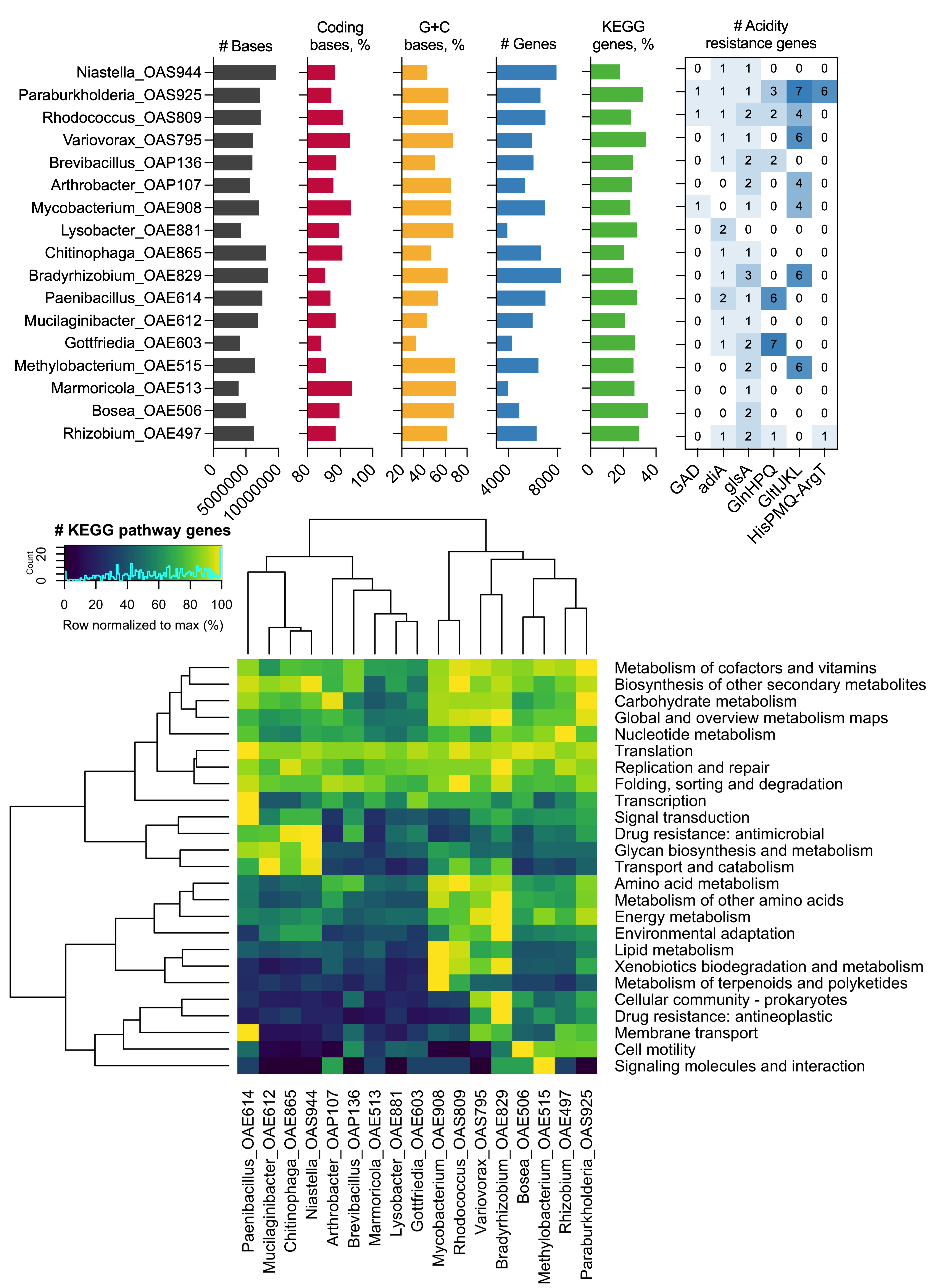

Supplement: S8 Fig — Bar graphs show genome characteristics (from left: the abundance of bases, coding, G + C bases, total genes, and KEGG pathway genes. The search for acid resistance system genes included GAD (EC 4.1.1.15 glutamate decarboxylase), AdiA (EC 4.1.1.19 arginine decarboxylase), glsA (EC 3.5.1.2 glutaminase), GlnHPQ (glutamine ABC transporter), GltIJKL (glutamate/aspartate ABC transporter), HisPMQ-ArgT (arginine/ornithine ABC transporter). The heat map shows normalized gene abundance for selected KEGG pathways. The data underlying this figure can be found at https://doi.org/10.6084/m9.figshare.26401315. (TIFF) [file pbio.3003358.s008.tiff]

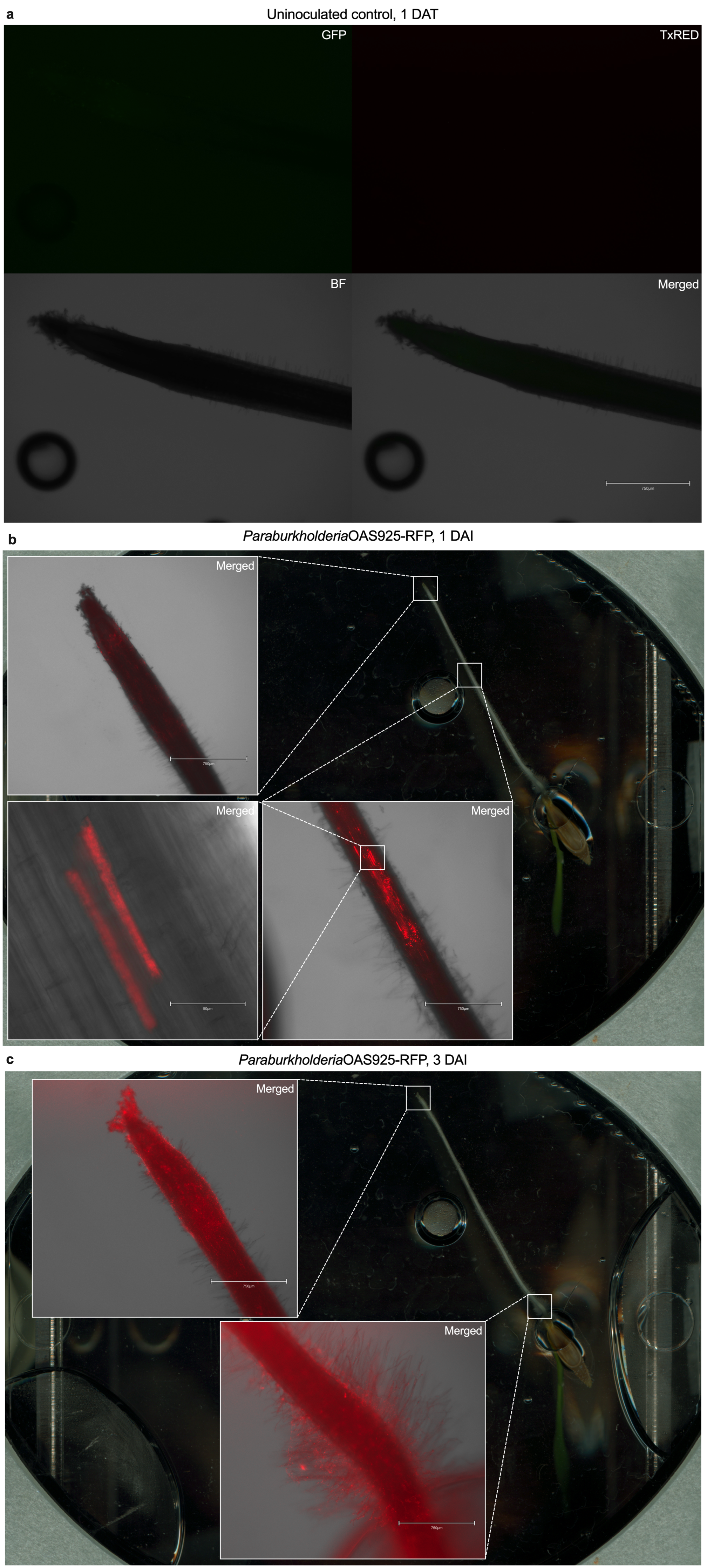

Supplement: S9 Fig — We inoculated RFP-expressing Paraburkholderia sp. OAS925 into the B. distachyon rhizosphere in EcoFAB 2.0 devices on the day of transfer (DAT) for the seedling. The plots show (a) uninoculated plant control (1 DAT) and medium-inoculated plants (OD600 0.01) at (b) 1 and (c) 3 days after inoculation (DAI). EcoFAB 2.0 root scans indicate the locations for microscopy. The inset microscopy images show merged TxRed and bright-field (BF) channels. The microscopy images can be found at https://doi.org/10.6084/m9.figshare.26449852. (TIFF) [file pbio.3003358.s009.tiff]

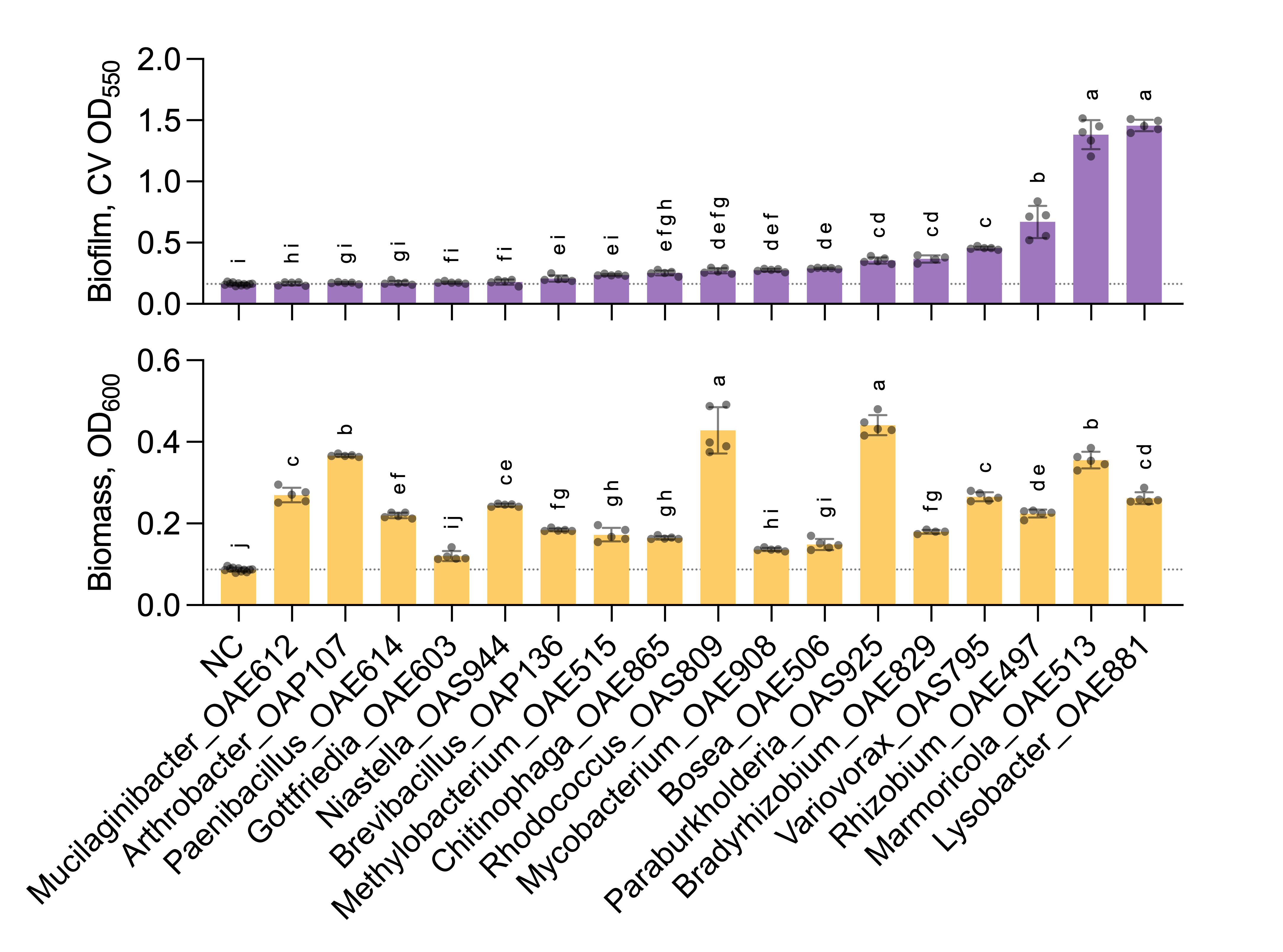

Supplement: S10 Fig — (Top) Biofilm was measured with crystal violet (CV) staining at OD550, and (Bottom) isolate growth was assessed based on OD600 values of isolates on the defined NLDM liquid medium for 3 days. The horizontal dotted line indicates the mean value of sterile medium negative control (NC). Different letters indicate statistically significant differences at p < 0.05, One-way ANOVA with Tukey’s test, n = 4–5 for isolates and n = 11 for NC. The data underlying this figure can be found at https://doi.org/10.6084/m9.figshare.26401315. (TIFF) [file pbio.3003358.s010.tiff]

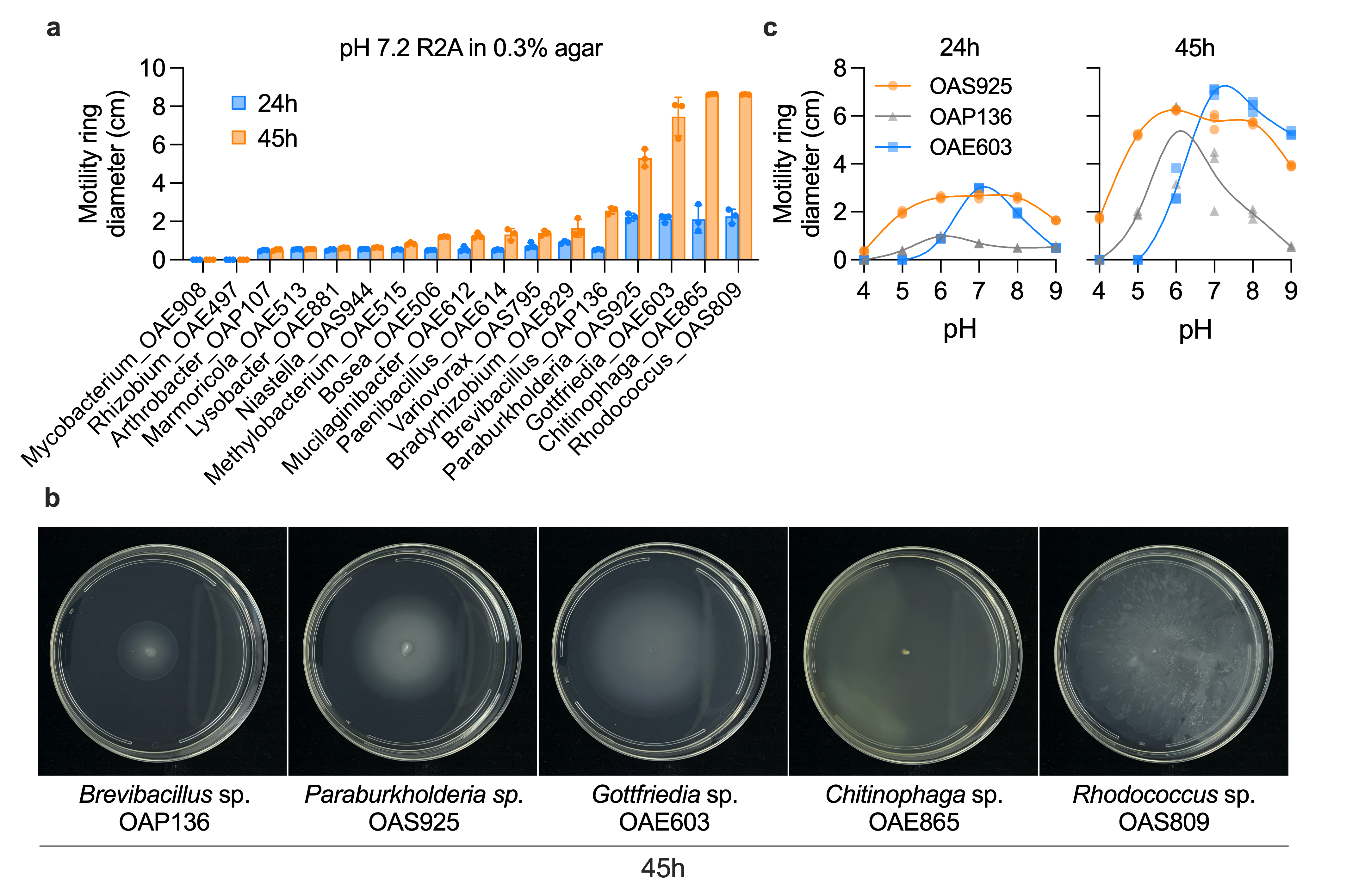

Supplement: S11 Fig — (a) The initial screen for swimming motility across bacterial isolates was measured 24 and 45 h after inoculation. (b) Phenotypes of the most motile strains at 45 h since inoculation. (c) pH effects on the motility ring diameter of isolates with bulls-eye colony morphology. Images of motility assays with bacterial isolates can be found at https://doi.org/10.6084/m9.figshare.26457928. The data underlying this figure can be found at https://doi.org/10.6084/m9.figshare.26401315. (TIFF) [file pbio.3003358.s011.tiff]
