# Supplementary material for: Breaking the reproducibility barrier with standardized protocols for plant–microbiome research
Source: PLoS Biol. 2025 Sep 8;23(9):e3003358. doi: 10.1371/journal.pbio.3003358 (PMC12416739; doi:10.1371/journal.pbio.3003358)
Supplement: Attachment — Submitted filename: Responses.pdf [file pbio.3003358.s026.pdf]

Thank you for the excellent comments that have greatly improved our manuscript. Please note that we are including two versions of the revised manuscript: (i) containing track changes to easily access edits and (ii) a clean version. **The lines in this response letter refer to the "track-changes" version.**

REVIEWERS' COMMENTS:

Reviewer #1:

This manuscript addresses the important and rarely-investigated issue of replicability of results between labs, specifically applied here to microbiome research. The organizers got together 5 participating labs and distributed an axenic growth chamber, pre-mixed microbial communities, plant seeds, and specific instructions to attempt to reproduce the same experiment as faithfully as possible. Much, but not all, of the experiment replicated. The paper is interesting, but should do some additional analyses on the results, particularly related to the differences that do exist between labs. The authors focused mainly on how Paraburkholderia completely overtook the community in all labs. To some extent, the detailed look at Paraburkholderia feels like a bit of a distraction from the main interesting points about reproducibility, because it is SOOO dominant. Focusing on this obscures other instructive differences, which I think should be more central. More specific comments follow, not in any particular order.

Thank you so much for recognizing the uniqueness of our study and finding it interesting. It will be of use to the community to recognize which data types might be difficult to reproduce. Thanks to your comments and the reviewer #2, we have revised the manuscript to provide additional data and in-depth statistical analyses of cross-laboratory variation in metabolite and microbiome data. We believe this successfully shifts attention to the main message of the story and also provides some interesting insights into sources of variation as described in more detail below.

The authors should model microbiome data including lab as a factor. How much of the variation in community in SynCom 16 is due to lab? How much remains unexplained? For community 17, after removing Paraburkholderia reads, what is left? There are unsatisfyingly few stats.

This is a great comment. We are now including detailed statistical analysis for rhizosphere metabolites and 16S-seq of root and media microbiome data. We have prioritized a unified approach to facilitate easy understanding for readers by utilizing ANOVA analysis for all datasets to estimate the main effects of labs vs. treatments. This includes comparison of significance levels (p-values using ANOVA, T-tests) and source of variation. Additionally, we present inter- and intra-laboratory coefficients of variation. These statistical results are presented in the new results section "Statistical evaluation of variation between labs", in new Figures S6 and S7, new Tables S7 and S8 and explained in M&M in section "Data management, statistical analyses, software, and data visualization". We also provide a corresponding paragraph in the discussion.

We are also providing raw read counts for microbiome data in a new table S3, which allows removal of specific SynCom members, e.g., Paraburkholderia.

Table S7: ANOVA results for rRNA sequencing data and metabolite intensity
Table S8: Pairwise results metabolites

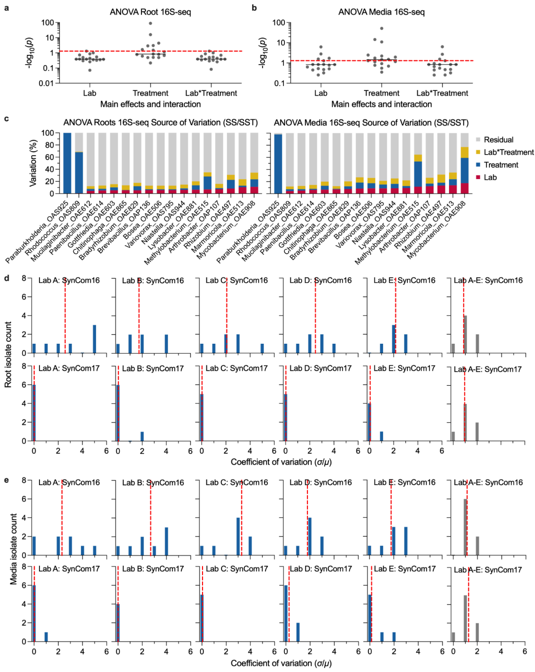

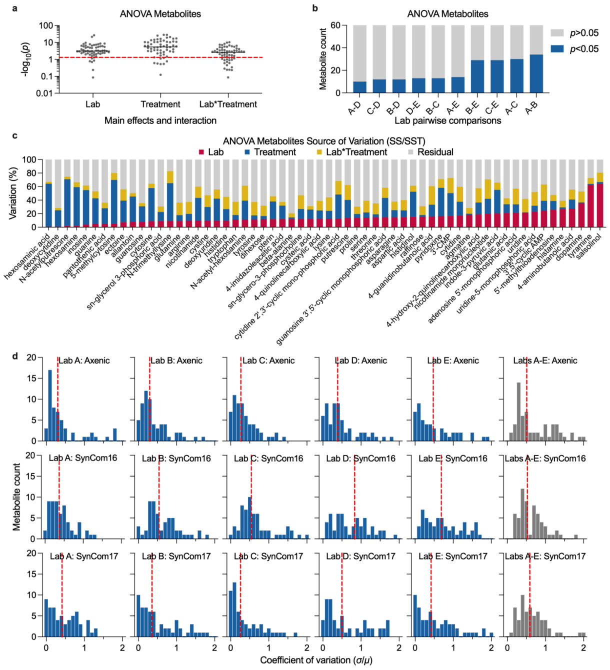

(Left) **S6 Fig**: Statistical analysis of 16S rRNA sequencing data. (a) ANOVA p-value of effects for root and media. Each point is a SynCom member with blue indicating significant values (red line p=0.05). (b) ANOVA source of variation for root and media calculated from ratio of squares (SS) for each effect and total sum of squares (SST). (c) Coefficient of variation (CV=σ/μ) distribution (median in red) for treatments within (blue) and across (gray) labs for root and media.

(Right) **S7 Fig**: Statistical analysis of 60 identified rhizosphere metabolites. (a) ANOVA p-value of effects for metabolite peak heights. Each point is a metabolite with blue indicating significant values (red line p=0.05). (b) Number of statistically different metabolites (p<0.05) in lab pairwise comparisons. (c) ANOVA source of variation for root and media calculated from ratio of squares (SS) for each effect and total sum of squares (SST). (d) Coefficient of variation (CV=σ/μ) distribution (median in red) for treatments within (blue) and across (gray) labs.

A couple things are curious about the inoculum. First, it appears quite uneven - I suppose the 16 or 17 members were intended to be mixed quite evenly. Can this be further explained? Second, the inoculum seems to bear nearly no resemblance to the ultimate community. Even Paraburkholderia seems not to be present in any substantial amount in the inoculum. Can the authors please present a table of percentages (in supplemental would be OK) that lists the median % of each community member in the inoculum and also in the ultimate community?

This is a good point–thank you. We certainly made every effort to be consistent with the inocula. As requested, we have now provided a Table with the total read counts with

min/max/median values and resulting relative abundance of microbes for all replicates (Table S3). It shows that there were reads for Paraburkholderia.

While we aimed to create equally mixed inoculum (1:1 cells), the DNA extraction efficiency and PCR amplification can differ between microbes. Additionally, the abundance in the 16S rRNA data is biased due to the number of copies of the 16S rRNA gene in each of the isolates.

We are now including how the data were normalized in M&M:

L492-494: “The relative abundance of each SynCom member was calculated as the proportion of total microbial reads (excluding plant reads) per sample.”

Related to the inoculum, it is odd that the inoculum in Figure 3 (plants) appears different than the inoculum in figure S3 (media). Were the media and plant inoculations NOT done simultaneously, such that independent inocula batches were thawed for the media?

Thanks for your comment. It was an oversight not to explain this clearly in the Material and Methods section. Each of the two SynCom inocula (17 and 16-member) was done in one batch and since the analyses were performed on two components of the same EcoFAB (plant vs bulk media) the inoculants were indeed consistent. However, since the microbial abundances were lower in the media than in the plant tissue, those samples required additional amplification cycles in order to generate enough product for sequencing. Since two different amplification cycles were used for each of these sample types, we also subjected the inoculants to each amplification protocol in order to identify how the cycle numbers might have impacted the data. For your convenience below is a figure incorporating the number of amplification cycles to clarify that these reflect the preparation of the different sample types (30 PCR cycles for Media and 22 cycles for Root to achieve similar DNA yield). Let us know if you see a value in including it in the manuscript. We believe that a simple statement in M&M should be sufficient:

L478-480: To allow direct comparison, the SynCom16 and SynCom17 inocula were each amplified for both 22 and 30 PCR cycles, mirroring the amplification conditions used for root and media samples, respectively.

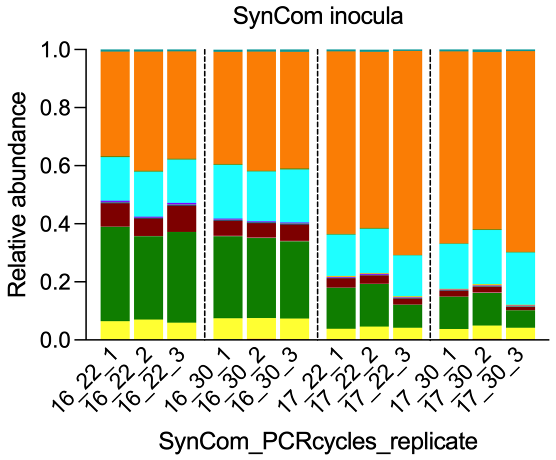

Particularly the media in Fig. S3, there are visible bars of "unknown" sequences colored black. What is the diversity of "unknown" in this? For example, if the "unknown" is all the same thing, isn't that a clear case of contamination? And if it's a mix of different things, how to explain it?

Good catch and this is something we should address in the manuscript. We have conducted BLAST search on these unknowns and this suggests that they are several human-associated microbes, which suggest handling as their potential source. For context, these media samples had to be amplified 32 PCR cycles and the templates were very dilute. This is now addressed in the table and result text and an associated M&M section.

Results L172-174: “The three samples with the highest proportion of unknown reads (>2.5%) contained OTUs associated with human microbes (Table S4), suggesting introduction during handling.”

M&M L490-492: “The three samples with the most unknown reads were analyzed via NCBI Microbial Nucleotide BLAST with Representative Genomes database and MegaBLAST algorithm to identify potential matches.”

S4 Table: 16S rRNA sequencing read counts in media and root samples

OTU	LabC-Media_13_16Syn	LabD-Media_12_16Syn	LabD-Media_13_16Syn	Seq	Top score hits in BLASTN search at NCBI - the most frequent genera [number of hits]
OTU32		7	188	CCGTTCGCTAC	NA
OTU72			37	CCCTTTGCTCC	Ancylomarina [1]
OTU33	152		2	GTTTGATCCCC	Staphlococcus [24]
OTU18	145	124	326	CTGTTTGCTCC	Actinobacter [3]
OTU51	3	36	1	TTTGCTCCCCA	Acinetobacter [23]
OTU61	65		14	GTTTGCTCCCC	Pseudomonas [17], Phytopseudomonas [6]
OTU20	375			GTTTGCTCCCC	Rahnella [12], Yersinia [4], Rouxiella [4], Serratia [3], Chimaeribacter [2], Ewingella [2]
OTU128			6	CTGTTTGCTCC	Labilithrix [1]
OTU28	243			GTTTGATACCC	Ihuprevotella [2]
OTU31	456			GTTTGATCCCC	Lacicoccus [2], Salinicoccus [1]
OTU38			8	CTGTTTGCTCC	Streptococcus [24]
OTU19	378			GTTTGCTCCCC	Clostridium [3]
OTU62	67			GTTTGCTACCC	Desulfovermiculus [1]
OTU46			107	CTGTTTGCTCC	Niallia [5]
SUM	1884	167	689		

For some analysis (eg microscopy) was done in the main host lab. I think it is worth identifying which of the labs that was. Those analysis could mention, for example, that microscropy was conducted on plants grown in Lab A.

This is a great suggestion. We have identified the lab in the microscopy, motility and biofilm formation assays in the figure captions and M&M section.

caption of S9 Fig: “conducted on plants grown in the lab A”
caption of S10 Fig: “... by the lab A”
caption of S11 Fig: “...in the lab A”

L497: “Lab A conducted the biofilm formation assays.”
L547: “Lab A conducted the plant growth and microscopy. “
L566: “Lab A conducted the motility assays.”

In Figure S1, each column (group of measurements of the same phenotype) should have the same Y axis so that the plots can be compared. And I think it's further preferable that columns and rows are switched, such that one can look across from left to right and see the shoot dry weight in each lab, for example.

This is an excellent suggestion, we have implemented the change by adjusting the Y axis and swapping the order of the plots. We have also simplified the design by removing repeating axis labels to make cross-laboratory comparisons easier.

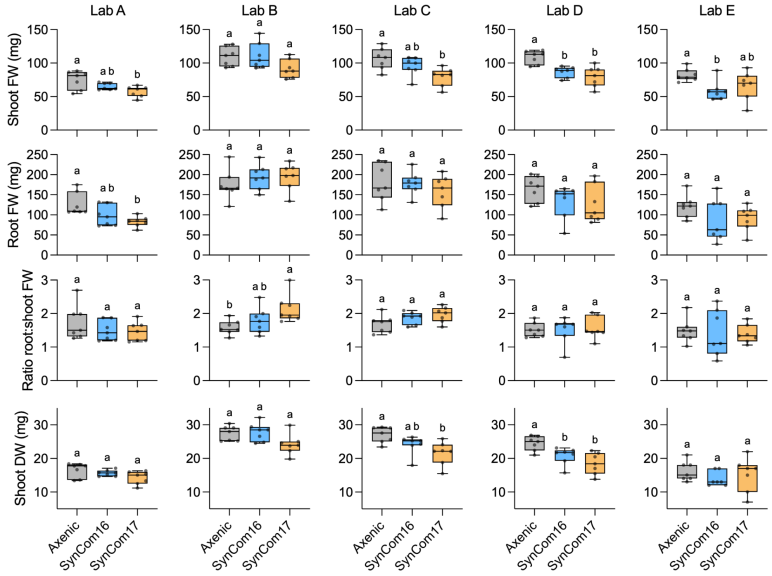

Likewise in Figure S2, the Y axes should be made comparable. The temperature side is OK, but the light levels are highly variable across labs and this should be immediately apparent looking at the graphs, but it's somewhat hidden due to the variable axes.

Thank you for pointing this out. We have adjusted the panel (b) caption: “Normalized illuminance (% of lab maximum value)”.

We have specified in M&M the procedure: L397-399: “ Each lab used two data loggers (HOBO MX2202 and UA-002-64) to track illuminance in lux normalized to maximum value per lab (for dark duration confirmation) and temperature.”

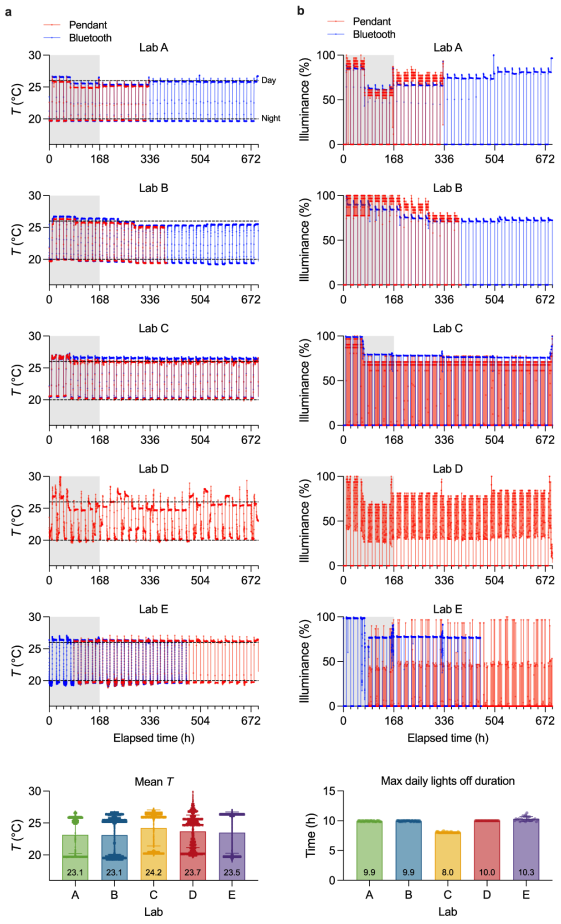

Fig. 2A is confusing. The barplots look like this is a continuous distribution, but as I understand it, if a device was sterile, it gets a grey box, and if it's contaminated, it gets a black box. It would be clearer if the boxes were separated so they could be seen as discrete data points, or even I think it would be clearer if the Y axis showed # of contaminated boxes and most are 0 and those that got contaminated go to 1. For example. But it's confusing as is.

Thanks for the excellent suggestion. We have changed the figure 2a according to your advice:

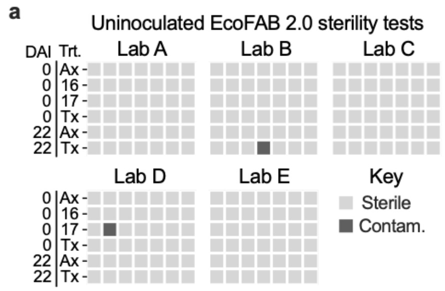

We have also adjusted the figure caption: "(a) Sterility of uninoculated EcoFAB 2.0 devices tested across laboratories A-E and treatments (Trt.: Ax-Axenic, 16-SynCom16, 17-SynCom17, Tx-Medium technical control) at day 0 and day 22 after inoculation (DAI). The medium from these devices was incubated on LB agar plates for 22 days to observe bacterial

colony formation. Each square represents one test with gray fill indicating sterility and black contamination. “

in Fig 2C, there are some differences between the labs in terms of measurement times. Lab B took a measurement at 4 days? Lab E did 0 days but skipped 14 days? What happened here, a miscommunication or technical failure or something?

Some laboratories had to adjust their scanning timepoints based on scanner availability. However, all of them fulfilled the requirement of providing scans from 3 timepoints. We are clarifying this in Results and M&M:

L125: “root imaging every seven days” was changed to “root imaging at three timepoints”

L407-409: “Evaporated water was re-supplied and roots imaged by scanning every seven days. Some laboratories shifted their root imaging timepoints based on scanner availability.”

Figure S4, putting each lab with a totally different NMDS2 plot is difficult (for me) to interpret. Is not it easier and more informative to simply use the combined lab plot to generate the spacings for the NMDS and simply erase the points from the other labs so that points from a single lab can be shown individually?

Excellent suggestion! We have done as you suggested. Please, see the improved figure below.

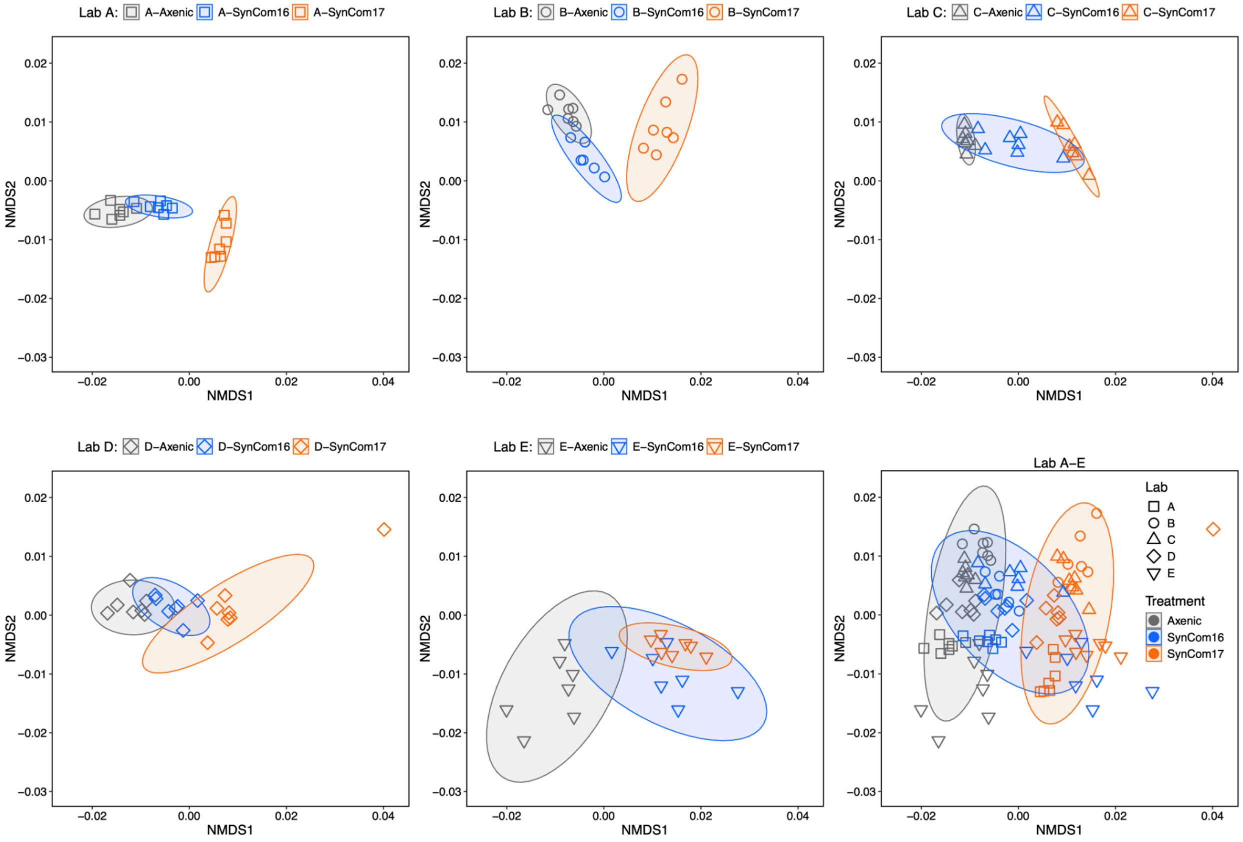

We have also matched the spacing in 16S sequencing NMDS figure to allow for a direct comparison between media and Root microbiome composition:

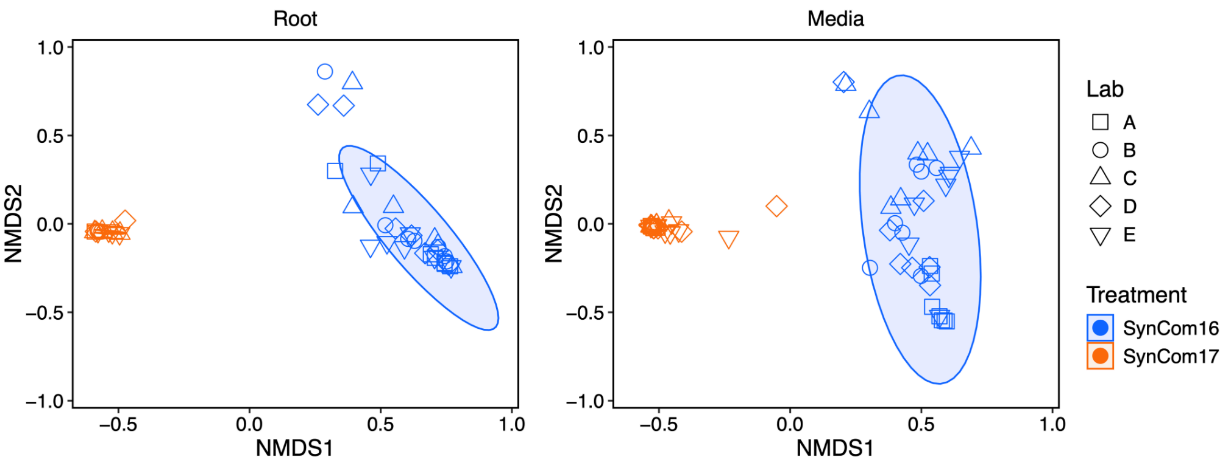

In the methods, please include the min/max/median sequencing depth for the microbiome samples.

Done. We now provide read counts with min/max/median for all sequencing samples in the Table S3.

Line 276.. the authors say the ecofab devices are "free". Surely this can't be true, or must have some limits. Please revise to clarify. It means free if one is part of a JGI user program? Is that limited to USA? Grant recipients of some form? Etc.

We agree that this is misleading–thank you for pointing this out. We have modified the statement as follows: L335-339: “The EcoFAB 2.0 devices (up to 50 pieces), B. distachyon Bd21-3 plant line, and metabolomics methods used in this study are currently freely available to domestic and international researchers via Joint Genome Institute (JGI) User Programs (https://jgi.doe.gov/), subject to a competitive scientific review processes.”

Reviewer #2 (Mengcen Wang):

This study addresses a critical challenge in microbiome research, inter-laboratory reproducibility, by assessing the consistency of synthetic community assembly experiments across multiple laboratories. The authors leverage ecosystems involving Brachypodium distachyon, two different synthetic bacterial communities, and sterile EcoFAB 2.0 devices, providing valuable insights into inoculum-dependent microbiome shifts and plant responses. While the study is generally well-structured and presents important findings, some areas require further clarification and improvement:

Dear Dr. Wang, we would like to thank you for recognizing the value of our study in addressing the challenges in reproducible microbiome research. We are grateful for your insightful comments that we have implemented as outlined below to improve the study and make it more clear.

1. It was indicated that the root system development was analyzed using RhizoNet (Lab B-E) and ImageJ (Lab A). Why did these five laboratories not standardize their analytical methods to minimize variability and improve reproducibility? Furthermore, it would be beneficial to include representative images of root systems.

This is a good point, In hindsight it would have been beneficial to standardize the scanners, but we decided not to require participants to buy new equipment (scanners, growth chambers, etc). In the case of scanners, we frankly did not expect this to be a source of variation between labs. This was not the case and is one of the many things we learned as part of this study. We are adding the following sentence to the discussion:

L323-325: “Additionally, standardization of the scanner model for automated root system analysis could help reduce variability and improve reproducibility.”

As suggested, we have created a new figure showing the root scans.

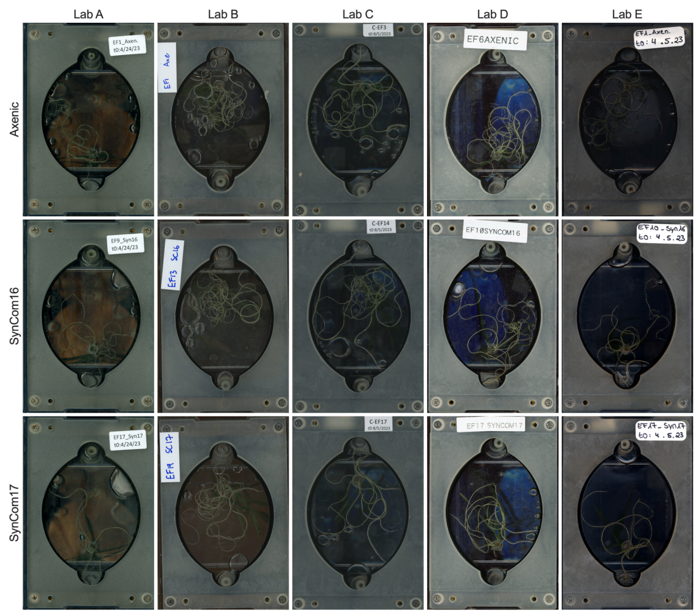

2. In line135-139, the uninoculated EcoFAB 2.0 sterility tests across laboratories A-E shown that the treatments of laboratory D in SynCom17 and laboratory B in medium-only control were contaminated. It would be more appropriate to redo the experiments to ensure the absence of contamination and further validate the reliability of the results.

Thank you for the observation. We believe that one of these was possibly a false positive due to a cracked lid in one of the plates. Despite this, the overall sterility rate was extremely high. We are not stating this in the discussion:
L260-263: “Of the 210 sterility tests performed across the 5 labs, only 2 potential contamination events were detected (Fig. 2a). One of these was from a plate with a cracked lid and so we conclude that the EcoFAB 2.0 devices and protocols were effectively able to achieve sterility across all labs.”

3. The SynCom17, which contained the dominating bacteria Paraburkholderia sp. OAS925 could reduce root growth of B. distachyon. How about the effects of the single strain Paraburkholderia sp. OAS925 on B. distachyon growth?

We agree that it would be interesting to assess the impacts of *Paraburkholderia* sp. OAS925 and other dominant isolates on plant growth. However, as you also point out (#5 below) given that there is a biomass decrease in both SynComs we think investigating this in more detail is beyond the scope of the current study. This is also consistent with Reviewer #1’s guidance that we should focus more on the multilab experiment vs. the Paraburkholderia. We now suggest these experiments as interesting future research directions

L310-313: “Additionally, future testing of the effects of Paraburkholderia sp. OAS925 and other dominant isolates on plant growth can provide additional insights into the extent that the observed growth effects are indeed a direct result of a single strain.”

4. L209-210 what does " its potential to outgrow competitors when cultured with common soil metabolites in the NLDM " mean?

Thank you, we have changed the sentence so it is easier to understand:
L242-246: *"Paraburkholderia* sp. OAS925 exhibited the sixth-highest biofilm formation and most liquid-culture growth (**Fig. S10**), highlighting its potential to outgrow many other bacteria in the SynCom17 [18]."

5. L226-L228, "This finding is consistent with a previous study showing Paraburkholderia sp. OAS925 dominance in the B. distachyon root and rhizosphere microbiota and decreased fresh root biomass." However, in Figure 2b, the shoot fresh weight (FW) of plants treated with both SynCom 16 and SynCom 17 was significantly reduced compared to axenic conditions. Moreover, no significant difference was observed between SynCom 16 and SynCom 17 treatments, suggesting that the SynCom 16 community, even without the addition of Paraburkholderia sp. OAS925, is sufficient to reduce shoot FW levels. Therefore, this does not conclusively demonstrate that the reduction in shoot FW under SynCom 17 treatment is specifically driven by Paraburkholderia sp. OAS925.

This is another excellent point. You are right that both SynCom treatments reduced the biomass (though the magnitude of SynCom17 was larger).

This has now been clarified in the revised text: L146: "When plant biomass data were combined across laboratories, we observed a significant decrease in shoot fresh weight and dry weight of plants inoculated with SynCom17, and to a lesser extent SynCom16, relative to the axenic treatment (Fig. 2b)."

We have also revised the discussion text by omitting the biomass influence and moving it to another paragraph to improve the discussion flow.
L283-284: "Our study confirms previous findings that *Paraburkholderia* sp. OAS925 dominates the root microbiome of *B. distachyon* when part of the SynCom [11]"

To reflect your point further, we have revised discussion paragraph to suggest that the plant growth inhibition could be caused by other bacteria:
LX301-305: "Both SynCom16 and SynCom17 decreased plant biomass, with SynCom17 having a more pronounced effect (Fig. 2, Fig. S1). The observed dominant colonization by *Paraburkholderia* (Fig. S6, Video S1 and Video S2) or other bacteria might disrupt plant nutrient homeostasis, as the root microbiome plays a crucial role in forming root diffusion barriers and maintaining plant mineral nutrient balance [5]."

6. In L303, the manuscript does not clearly explain the rationale behind the 1:1 ratio of strains in the SynComs. It is recommended to provide additional explanation to enhance the scientific rigor and credibility of the study.

This is a great question and is something that we debated when deciding on the experimental design. In the end we decided to use the simplest approach which is equal ratios as used in the seminal papers by Castrillo et, Duran et, and recently Coker et al.. We have added this to the manuscript as described below:

Line 370-371. “We used SynComs composed of equal numbers of cells for the constituent strains, which is a widely used approach (Coker et al. 2022; Castrillo et al. 2017; Durán et al. 2018).”

7. In L362, the authors indicate that the hydroponic medium sample was used as the root exudate for metabolomic analysis. But how did they rule out potential interference from bacterial metabolites in the medium?

This is a good point. The medium samples from SynCom inoculated treatments represent a result of both plant and bacterial metabolism. For this reason, we have changed “root exudates” in figure S5 caption and M&M to “rhizosphere metabolites”. Additionally, in the text we also talk about “exometabolomes” to make this mixed effects clear.

8. The colonization of bacteria in B. distachyon roots can be influenced by various external factors. The assessment of bacterial motility on liquid soft agar only reflects swimming ability in liquid culture media and does not provide definitive evidence that Paraburkholderia sp. OAS925 can outcompete other members during SynCom colonization.

We fully agree that multiple traits, i.e., fast growth, motility, C utilization, low pH resistance, etc all can enable *Paraburkholderia* sp. OAS925 to outcompete other strains. To clarify this point, we have revised the text to include:

L288-293: “Our study suggests several traits that likely help contribute to the dominance of Paraburkholderia sp. OAS925 in SynCom17. For example its high motility in acidic environments (S11 Fig), such as the rhizosphere, might facilitate quick colonization of ecological niches and affect community assembly [24–26].

9. The analysis of microbial community and metabolome data is crucial for the research conclusions. However, it is not clear in the paper whether significance analysis was performed on this part of the data and what methods were used. If there is relevant analysis, please provide a complete description.

Thank you for your comment. In line with similar point by reviewer #1, we now include statistical significance analyses for microbial community and metabolome data that we present in a new result section 2.6 and describe in M&M.

10. It is recommended to present the analysis conclusions of some data in the supplementary information to enrich the content of the main text.

This a great point and one also recommended by Review #1. We have now included new SI Figures and added corresponding results and discussion sections which we feel provides a number of additional insights into the sources of variation in this multi-lab experiment (please see these under Reviewer #1 comments).
